# Extracellular vesicle-mediated pre-metastatic niche formation via altering host microenvironments

**DOI:** 10.3389/fimmu.2024.1367373

**Published:** 2024-03-01

**Authors:** Ying Li, Yan Zheng, Xiaojie Tan, Yongxing Du, Yingxin Wei, Shanglong Liu

**Affiliations:** ^1^ Department of Blood Transfusion, the Affiliated Hospital of Qingdao University, Qingdao, China; ^2^ Department of Operating Room, the Affiliated Hospital of Qingdao University, Qingdao, China; ^3^ Department of Gastrointestinal Surgery, the Affiliated Hospital of Qingdao University, Qingdao, China; ^4^ Department of Pancreatic and Gastric Surgery, National Cancer Center/Cancer Hospital, Chinese Academy of Medical Sciences and Peking Union Medical College, Beijing, China; ^5^ Department of General Surgery, Peking Union Medical College Hospital, Chinese Academy of Medical Science & Peking Union Medical College, Beijing, China

**Keywords:** pre-metastatic niches, extracellular vesicles, metastasis, immunosuppression, disseminated tumor cells

## Abstract

The disordered growth, invasion and metastasis of cancer are mainly attributed to bidirectional cell-cell interactions. Extracellular vesicles (EVs) secreted by cancer cells are involved in orchestrating the formation of pre-metastatic niches (PMNs). Tumor-derived EVs mediate bidirectional communication between tumor and stromal cells in local and distant microenvironments. EVs carrying mRNAs, small RNAs, microRNAs, DNA fragments, proteins and metabolites determine metastatic organotropism, enhance angiogenesis, modulate stroma cell phenotypes, restructure the extracellular matrix, induce immunosuppression and modify the metabolic environment of organs. Evidence indicates that EVs educate stromal cells in secondary sites to establish metastasis-supportive microenvironments for seeding tumor cells. In this review, we provide a comprehensive overview of PMN formation and the underlying mechanisms mediated by EVs. Potential approaches to inhibit cancer metastasis by inhibiting the formation of PMNs are also presented.

## Introduction

Cancer is a genetic disease with characteristics of disordered growth, increased invasion and metastasis, promotion of angiogenesis, metabolic remodeling and immune suppression. Cancer progression is a multistep and complex process that leads mainly to the inactivation of the tumor suppressor gene and activation of the proto-oncogene (1). Increasing evidence shows that extracellular vesicles (EVs)-mediated bidirectional cell-to-cell communication is also involved in cancer development and progression (2). EVs with a broad size range are produced by various cell types and present in biological fluids, including blood, ascites, bile, urine, breast milk and bronchoalveolar lavage fluids. The amount and composition of EVs are influenced by the external microenvironment, such as pH, hypoxia, irradiation, cell activation and oxidative stress (3). EVs can be categorized into three major groups according to their sizes and biogenesis mechanisms: 1) exosomes, ranging from 30 to 150 nm in diameter; 2) microvesicles (MVs), large membrane vesicles of 150–1000 nm in diameter direct budding from the plasma membrane; 3) apoptotic bodies and oncosomes of 1000–5000 nm in diameter, which are newly identified cancer-derived EVs. EVs encapsulated with a phospho-lipid membrane contain biologically active molecules, including DNA, RNAs, lipids, proteins and metabolites that are important intercellular messengers. Smaller shed MVs, which are ~100 nm in diameter, have also been reported. Exosomes are intraluminal vesicles generated by the inward budding of the endosomal membrane during the differentiation of multivesicular endosomes. Exosomes exhibiting a cup-shaped structure carry cell-surface and soluble proteins from the extracellular milieu (4). The mechanisms underlying the biogenesis of exosomes involve ras-related protein GTPase Rab, tumor susceptibility gene 101, apoptosis-linked gene 2-interacting protein X, syndecan-1, ceramides, endosomal sorting complexes required for transport proteins, phospholipids, tetraspanins and sphingomyelinases (5). Microvesicles, first described as subcellular material derived from platelets, are formed by outward budding and fission of the plasma membrane. EV biogenesis depends on distinct cell types, physiological states, culture conditions and the genomic health of cells. Rädler et al. have summarized more than 75 articles and discussed their findings on the formation and composition of exosomes and microvesicles, revealing multiple pathways that may be stimulation- and/or cargo-dependent (6). Because of the overlap in size and composition and commonalities in their biogenesis pathways, it remains challenging to define the mutually exclusive properties of EVs. Efforts to explore the mechanisms underlying the targeting of different cargoes, the biogenesis of vesicles and their role in different target cells are being undertaken to clarify the respective roles of the different EV types (7). EVs are stable in circulation, possess immune avoidance properties and pass through biological barriers to reach various sites because of their natural nanoparticle characteristics (8). EVs can protect cargo, and surface proteins in the membrane of EVs prolong circulation times and facilitate accumulation in specific organs. Currently, differential centrifugation is a common method to isolate EVs. However, this method cannot isolate pure populations of EVs because of their diverse sizes, similar morphology and variable composition. Thus, available centrifugal protocols to isolate EVs from samples yield heterogeneous vesicle populations. Other promising methods to purify specific EVs include discontinuous cushion gradients, membrane affinity columns, precipitation, size exclusion chromatography, filtration immunoaffinity capture and microfluidic systems (9). Rapid progress in methods to isolate EVs has increased our understanding of the biological functions of various EV types (10). However, validating their heterogeneity remains challenging because of technological shortcomings and biological knowledge gaps, thus limiting the widespread use of EVs. Therefore, novel methods are needed to explore the respective functions of the different types of EVs and to develop a classification and terminology system (11). Cells release EVs to communicate with other cells and affect the behavior of recipient cells under both physiological and pathological conditions (12). The abundance and composition of EVs differ between tumor-derived and normal cell-derived EVs. Cancer cells generate more EVs with distinct biological cargo than nonmalignant cells. As a cell-derived material, the various origins of EVs can affect target cells differently. Therefore, increased EV levels can distinguish individuals with cancer from those suffering from nonmalignant diseases. Tumor-derived EVs exhibit altered expression of proteins, miRNAs, mRNAs or mutant DNA alleles, which promotes metastasis by angiogenesis and induces matrix remodeling and metastatic niche formation (13). EVs discussed throughout this review refer to EVs originating from tumor cells. Tumor-derived EVs mediate bidirectional communication between tumor and stromal cells in local and distant microenvironments. Tumor-derived EVs activate stromal fibroblasts to differentiate into cancer-associated fibroblasts (CAFs), which supports tumor growth, stimulates endothelial cells to increase angiogenesis and induces the suppression of the antitumor immune response, thereby generating a favorable pre-metastatic microenvironment (14). Adaptation to the distant microenvironment is critical for metastasizing at different sites or organs. Accumulating evidence indicates that EVs are released from primary cancer cells and orchestrate the formation of pre-metastatic niches (PMNs) by transforming stromal cells to establish metastasis-supportive microenvironments (15). However, the biological functions of EVs and their underlying mechanisms on PMN formation remain unclear and warrant further investigation. In this review, the roles of EVs in PMN formation are discussed. We then describe how EVs regulate PMN formation by enhancing angiogenesis and vascular permeability, modulating stromal reprogramming and inducing antitumor immune suppression. Lastly, we discuss the clinical applications of EVs in cancer-targeted therapy.

## The role of EVs in PMN formation

PMNs are predetermined microenvironments that cancer cells establish to support the outgrowth of tumor cells in other organs before the arrival of circulating tumor cells (CTCs) (16). Both tumor-secreted factors and tumor-shed EVs contribute to the evolution of PMNs. PMNs are tumor growth-conductive microenvironments absent of cancer cells, which differ from the metastatic niche reprogrammed after the arrival of CTCs. The “seed and soil” hypothesis is defined as the primary tumor (the “seed”) preparing the local microenvironment in distant organs (the “soil”) where the microenvironment is hospitable for disseminating cancer cells to colonize. The crosstalk between cancer-released components (cytokines and EVs) and the local stromal microenvironment of the host is crucial for PMN formation. Vascular endothelial growth factor (VEGF)-A, placental growth factor, tumor necrosis factor α (TNF-α) and transforming growth factor β (TGF-β), which are induced by inflammation and hypoxia, are involved in developing PMNs (17, 18). Hypoxia-inducible factor-1 promotes granulocyte colony-stimulating factor (G-CSF) production by activating the NF-kB-G-CSF axis in breast cancer, inducing the recruitment of granulocytic myeloid-derived suppressor cells (MDSCs) to form PMNs in the lung (19). CCL2 from primary tumors induces the recruitment of Ly-6C (+) monocytes, regulatory T (Treg) cells and tumor-associated macrophages (TAMs) to facilitate the invasion of CTCs into PMNs for colonization at distant sites (20).

As potential mediators of cell-cell interactions, tumor-derived EVs that carry mRNAs, small RNAs, microRNAs, DNA fragments, proteins and metabolites circulate from their primary tumor to enable PMN formation in other organs. For example, uptake of pancreatic cancer-derived exosomes that enrich migration inhibitory factor (MIF) by Kupffer cells (KCs) increases the release of TGF-β, which, in turn, activate hepatic stellate cells (HSCs) to enhance fibronectin production, finally leading to the arrest of macrophages and neutrophils in the liver and PMN formation (21). Exosomes containing miR-100-5p, miR-21-5p and miR-139-5p from prostate cancer modify PMNs by increasing the expression of metalloproteinases (MMPs) and fibroblast migration (22). Brain astrocyte-derived exosomes transfer PTEN-targeting miRNA-19a to metastatic tumor cells, which reduces PTEN expression. This reduction leads to an increase in the secretion of CCL2 that recruits ionized calcium-binding adaptor molecule 1-expressing myeloid cells to enhance the proliferation of metastatic tumor cells in the brain (23). MVs from breast cancer induce bone-marrow-derived pro-angiogenic cells (BMDCs) infiltration and tumor colonization via osteopontin expression (24). Moreover, tumor-derived EVs carrying nucleic acid components significantly affect the behavior and genetic status of recipient cells. Medulloblastoma cell-derived MVs contain high levels of DNA fragments and amplify the oncogene c-Myc (25). These findings suggest that distant sites can be reprogrammed into tumor-promoting microenvironments by tumor-derived EVs, and the underlying mechanism depends on secondary sites, tumor types and their modes of interaction.

## Tumor-derived EVS determine metastatic organotropism (liver, lung, brain and bone)

**Figure 1:**
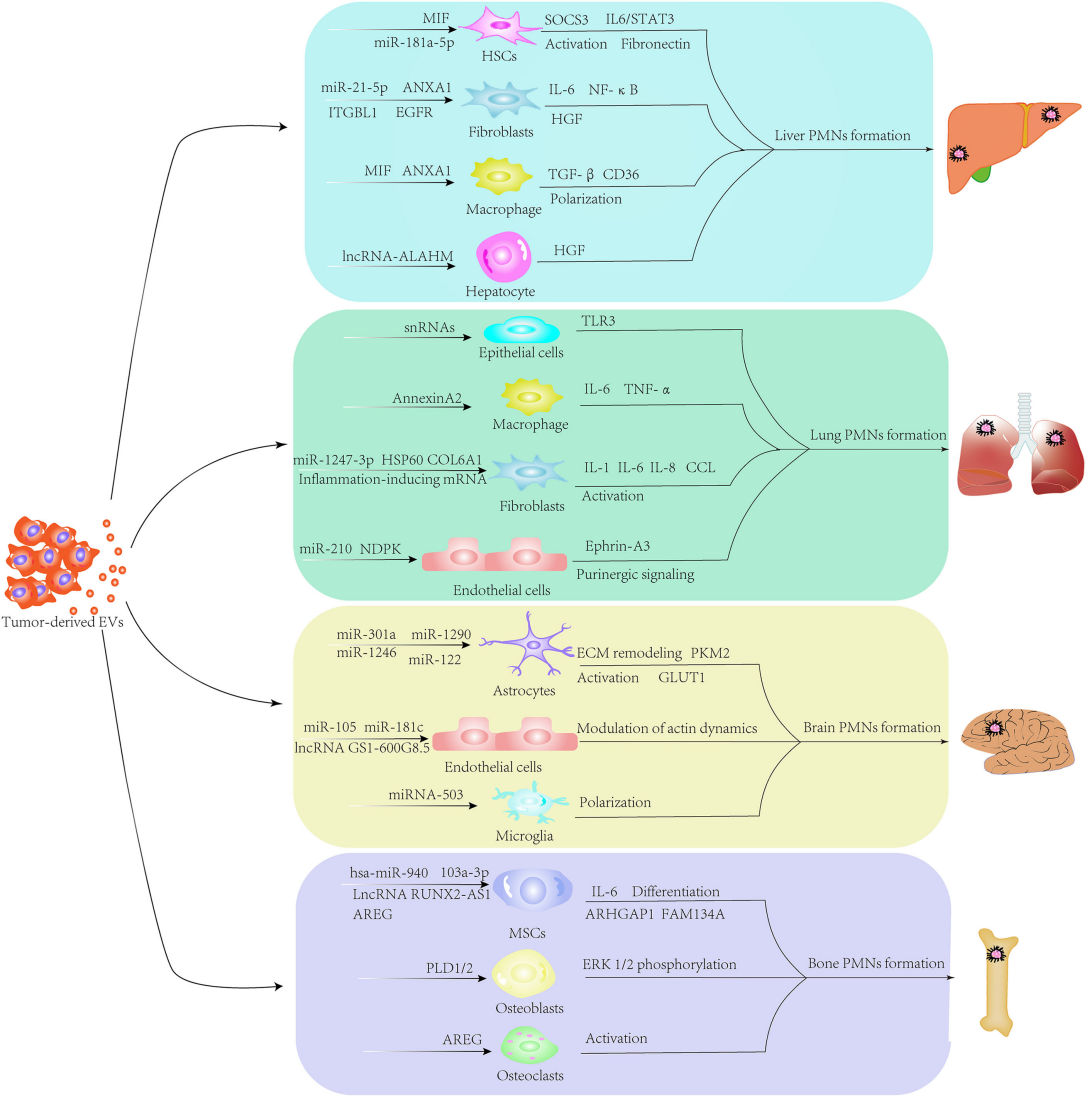
Formation of extracellular vesicle (EV)-mediated pre-metastatic niches. Through various signaling pathways, EVs secreted by primary tumor cells circulate to distant sites and remodel the local stromal cells in the pre-metastatic microenvironment. EVs carrying mRNAs, small RNAs, microRNAs, DNA fragments, proteins and metabolites educate stromal components in secondary sites to establish metastasis-supportive microenvironments for colonizing disseminated tumor cells.

Metastasis involves multiple steps, including vascular remodeling for cancer cell dissemination and circulation, formation of PMNs and colonizing primary cancer cells in distant organs, indicating that interactions between cancer cells and their target organs determine site-specific metastasis (26). The origin of primary tumors largely determines the pattern of metastatic organs. According to the “seed and soil” theory proposed by Stephen Paget, tumor metastasis to distant sites is not a random event but a process of tumor driving-organotropism (27). Certain primary tumors have a specific affinity for specific organs, and this specificity dictates the metastatic neoplasia at distant sites. For example, liver metastasis is often observed in colorectal cancers, gastric cancer, pancreatic cancer and breast cancer, whereas the most frequent primary tumors metastasizing to the lung include gastrointestinal tumors, breast cancer, renal carcinoma and bladder cancer. Prostate cancer frequently metastasizes to bone (28–30). Exploring the mechanisms that regulate organ-specific metastasis is crucial in designing drugs for target therapy (31). EVs target recipient cells via different mechanisms, including docking on the surface receptors and entering cells through endocytosis or directly fusing to the plasma membrane. These EV-cell interactions subsequently activate signal transduction or cargo transfer to target cells. Integrin expression profiles are linked to tissue organotropism. Integrins of EVs determine the organotropism at distinct organs, including the lung, liver, brain and bone. In detail, integrin αvβ5 is primarily expressed in liver-tropic exosomes, inducing liver tropism through enhancing adhesion to KCs. Integrins α6β1 and α6β4 are present in lung-tropic exosomes, resulting in lung tropism via stimulating adhesion of tumor-derived EVs to lung epithelial cells and fibroblasts and increasing exosome uptake in the lung. Integrin Gβ3 is detected in exosomes derived from brain-tropic cells, leading to brain metastasis by interacting mainly with CD31-positive brain endothelial cells. Integrins also interact with extracellular matrix (ECM) components that contribute to the internalization of EVs in specific organs. Moreover, exosomal integrins induce proto-oncogene tyrosine-protein kinase Src and enhance the expression of promigratory and pro-inflammatory S100 genes, promoting PMN formation and facilitating tumor metastasis (32). Recent developments revealed that EVs, as the metastatic organotropism drivers, build local and distal microenvironments conducive to tumor metastasis (**Figure 1**). Mo et al. demonstrated the critical role of tumor-derived EVs in organotropic metastasis by modulating the formation of PMNs (33). EVs released from primary tumor cells are internalized by resident cells when arriving at PMNs, transforming the distant microenvironment into a hospitable “soil” for cancer cells to colonize and proliferate. The organotropism of EV biodistribution is determined by multiple factors, including the metastatic properties of primary tumors, cargoes, membrane proteins and the tumor microenvironment (TME) (34). Distant metastasis, including in the lungs, liver, brain and bone, mediated by EVs has been investigated recently (**Table 1**).

**Table 1:** Summary of studies on EVs from tumor mediated metastatic organotropism.

Secondary sites	Donor cells	Cargo	Target cells	Specific mechanism	Refs.
Liver	CRC	miR-181a-5p	HSCs	Activating SOCS3 and IL6/STAT3 pathway	(26)
	CRC	miR-21-5p	Macrophages	Producing inflammatory cytokines through TLR7	(27)
	CRC	CD63 and HSP70	Stromal cells	Promoting metastasis via SDF-1α/CXCR4 axis	(28)
	CRC	ITGBL1	Fibroblasts	Activating NF-κB pathway	(29)
	PDAC	MIF	Kupffer cells	Increasing TGF-β secretion	(13)
	PDAC	MIF	HSCs	Upregulating fibronectin production	(13)
	PDAC	Intravesicular cargo	Macrophages	CD36 promotes internalization of tumor microvesicles	(30)
	PDAC	Annexin A1	Macrophages	Polarizing the macrophage to M2 phenotype	(31)
	Gastric cancer	EGFR	Stromal cells	Enhancing HGF expression via inhibiting miR-26a/b	(32)
	Lung cancer	LncRNA-ALAHM	Hepatocyte	Increase HGF expression	(33)
Lung	Melanoma cells	snRNAs	Lung epithelial cells	Inducing chemokine production and neutrophil recruitment via activating lung epithelial TLR3	(34)
	Breast cancer	AnnexinA2	Macrophage	Increasing IL-6 and TNF- via activating p38MAPK, NF-κB, and STAT3	(35)
	Breast cancer	HSP60	Lung fibroblasts	Recruiting monocytes and suppressing T cell via TLR2-MyD88-NF-κB	(36)
	HCC	miR-1247-3p	Fibroblasts	Enhancing pro-inflammatory cytokines by β1-integrin–NF-κB	(37)
	Melanoma	Inflammation-inducing mRNA	Fibroblasts	Enhancing expression of pro-inflammatory cytokines and chemokines	(38)
	HCC	miR-103	Endothelial cells	Disrupting integrity of endothelial junction by targeting endothelial junction proteins.	(39)
	Breast cancer	miR-210	Endothelial cells	Enhancing angiogenesis and increase vascular permeability	(40)
	osteosarcoma	COL6A1	Fibroblasts	Activating fibroblasts by secreting IL-6 and IL-8.	(41)
	CAFs	Integrin α2β1	Fibroblasts	Activating fibroblasts through TGF-β and inducing extracellular matrix remodeling	(42)
Brain	Breast cancer	miR-181c	Endothelial cells	Degrading PDPK1 in endothelial cells, leading to BBB destruction	(43)
	Breast cancer	miR-105	Endothelial cells	Increasing vascular permeability by reducing the tight junction protein ZO-1	(44)
	Breast cancer	LncRNA GS1-600G8.5	Endothelial cells	Promoting DTCs to cross the BBB system	(45)
	Breast cancer	miR-301a	Astrocytes	Altering the expression of ECM-remodeling proteins by downregulating TIMP-2	(46)
	Breast cancer	miR-1290 and miR-1246	Astrocytes	Promoting breast cancer stemness and brain metastasis through the FOXA2-CNTF	(47)
	Breast cancer	miR-503	Microglia	Triggering M1–M2 polarization of microglia through STAT3 and NF-κB	(48)
	Breast cancer	miR-122	Astrocytes	Suppressing glucose uptake by astrocytes via downregulating PKM2 and GLUT1	(49)
Bone	Myeloma	miR-103a-3p	BMSCs	Inhibiting osteogenesis and increasing IL-6 secretion	(50)
	Myeloma	LncRNA RUNX2-AS1	MSCs	Inhibiting osteogenic differentiation via lncRUNX2-AS1/Runx2	(51)
	Multiple myeloma	AREG	MSCs	Blocking osteoblast differentiation by activating EGFR and releasing IL-8	(52)
	Lung cancer	AREG	Pre-osteoclast	Increasing the expression of markers of osteoclastogenesis by activating EGFR	(53)
	Lung cancer	miR-21	Bone marrow monocyte	Targeting PDCD4 to facilitate osteoclastogenesis	(54)
	Breast cancer	Hsa-miR-940	MSCs	Promoting osteogenic differentiation of MSCs through targeting ARHGAP1 and FAM134A	(55)
	Prostate cancer	RANK ligand	Osteoclast	Promoting osteoclast differentiation by stimulating NF-κB activity	(56)
	Prostate cancer	PLD1/2	Osteoclast	Activating osteoblast differentiation by enhancing ERK 1/2 phosphorylation,	(57)

### Liver metastasis

Liver metastasis is the most common issue observed in various types of cancer. Several studies have confirmed the role of cancer cell-derived EVs in establishing PMNs in the liver. Zhao et al. demonstrated that EVs released from colorectal cancer (CRC) containing miR-181a-5p persistently activated HSCs through the SOCS3 and IL6/STAT3 signaling pathways. EVs-miR-181a-5p derived from CRC activates HSCs to remodel the TME for circulating tumor cells to readily colonize and proliferate (35). EVs enriched in miR-21-5p secreted from CRC polarize and promote macrophages to produce inflammatory cytokines such as interleukin-6 (IL-6) through Toll-like receptor 7 (TLR7), creating PMNs for liver metastasis (36). Exosomes from CRC increase the metastatic tumor distribution in the liver by recruiting C-X-C chemokine receptor type 4 (CXCR4)-expressing stromal cells to develop a pre-metastatic microenvironment (37). Integrin beta-like 1 (ITGBL1)-rich EVs from CRC activate fibroblasts to promote liver metastasis via the NF-κB pathway. Jianpi Jiedu Recipe (JPJDR), a traditional Chinese medicine, effectively prevents CRC liver metastasis by reducing ITGBL1-rich EVs secretion and suppresses fibroblast activation through the ITGBL1-TNFAIP3-NF-κB signaling pathway (38). Costa-Silva et al. showed that EVs from pancreatic ductal adenocarcinoma (PDAC) containing high levels of MIF increase transforming growth factor β secretion by KCs and upregulate fibronectin production in HSCs (21). Pfeiler et al. found that the intravesicular cargo of MVs from PDAC was transferred to macrophages in a CD36-dependent manner to support long-term reprogramming for liver metastasis (39). Annexin A1 contained in EVs from pancreatic cancer polarizes macrophages to the M2 phenotype in TME, activating endothelial cells and fibroblasts to increase angiogenesis and matrix remodeling (40). Additionally, EGFR in exosomes secreted from gastric cancer cells can transfer to liver stromal cells and enhance hepatocyte growth factor (HGF) via reducing miR-26a/b expression. Upregulated HGF in stromal cells ultimately binds to the c-MET receptor on CTCs, facilitating liver-specific metastasis (41). Highly enriched lncRNA-ALAHM in EVs from lung adenocarcinoma increases HGF expression in hepatocytes to promote lung cancer liver metastasis significantly (42). Cumulative evidence indicates that EVs establish PMNs and facilitate the development of liver metastasis by activating pro-inflammatory signaling pathways and modulating stromal cell activity.

### Lung metastasis

The lung is a frequent metastatic site for liver, colorectal, gastric, breast and pancreas cancers. Inflammation characterized by the infiltration of pro-inflammatory cytokines and inflammatory cells is an essential feature of PMNs. Liu et al. found that primary tumor-derived exosomes, enriched in small nuclear RNAs, activated TLR3 expression in lung epithelial cells and induced chemokines (CXCL1, CXCL2, CXCL5, and CXCL12) secretion. The accumulation of chemokines in the lung initiates neutrophil recruitment and immunosuppression, contributing to the formation of a pro-inflammatory microenvironment (58). Maji et al. revealed that exosomal-annexin A2 from breast cancer promoted macrophages to secrete IL-6 and TNF-α activation of the p38MAPK, NF-κB, and STAT3 pathways, resulting in the establishment of PMNs in brain and lung tissues (59). Heat shock protein 60 (HSP60) on the surface of EVs released from breast cancer stimulates CCL2 expression in lung fibroblasts via the TLR2-MyD88-NF-κB signal pathway, leading to monocyte recruitment and suppression of T cell function (43). By directly targeting β-1,4-galactosyltransferase III (B4GALT3), metastatic hepatocellular carcinoma (HCC) cells derived exosomal miR-1247-3p activate fibroblasts to secrete pro-inflammatory cytokines, including IL-6 and IL-8, through the β1-integrin–NF-κB pathway (44). In melanoma, EVs containing inflammation-inducing mRNA activate fibroblasts in the lung to enhance the expression of multiple pro-inflammatory cytokines and chemokines, including IL-1α, IL-1β, CXCL10, CXCL1, CCL2, CCL3 and CCL5 (45). In addition to activating a pro-inflammatory reaction, EVs are also directly involved in the vascular remodeling of the lung. Fang et al. showed that EV-packaged miR-103 from HCC cells promoted lung metastasis by targeting endothelial junction proteins. The integrity of the endothelial junction was disrupted by inhibiting the expression of vascular endothelial-cadherin (VE-cadherin), p120-catenin and zonula occludens-1 (ZO-1) when EVs containing miR-103 are delivered into endothelial cells (60). A study from Kosaka et al. revealed that miR-210 carried by EVs from breast cancer cells is transported into endothelial cells to enhance angiogenesis and increase vascular permeability by suppressing the expression of ephrin-A3 (46). EV-associated nucleoside diphosphate kinase A and B (NDPK) from breast cancer attenuates monolayer integrity and enhances vascular endothelial cell migration via purinergic signaling, contributing to host microenvironment plasticity and creating lung PMNs for metastatic outgrowth (47). Furthermore, stromal cell remodeling mediated by EVs plays a critical role in PMN formation in the lung. CAF-derived EVs participate in forming PMNs in a different manner to tumor EVs in the lung. EVs from primary tumor cells enable CAFs/myofibroblast differentiation and activation, which modify the microenvironment to be suitable for CTC colonization. Exosomal collagen type VI alpha 1 (COL6A1) contained in osteosarcoma (OS) cells induces the transfer of normal fibroblasts into CAFs by secreting IL-6 and IL-8. Interestingly, the activated CAFs promote the invasion and migration of OS cells by mediating the TGF-β/COL6A1 signaling pathway, forming a positive feedback loop (48). Kong et al. showed that CAF EVs in salivary gland adenoid cystic carcinoma activate lung fibroblasts through the TGF-β signaling pathway and induce lung fibroblasts to produce periostin, leading to enhanced ECM remodeling for establishing PMNs (49). Unfortunately, the mechanism underlying EV-mediated lung-specific metastasis remains unknown. According to Hoshino et al., tumor exosome integrins determine organ-specific metastasis. Integrins α6β4 and α6β1 enriched in exosomes are associated with lung metastasis, whereas exosomal integrin αvβ5 is linked to liver metastasis, indicating that exosomal integrins are effective predictors for organotropic metastasis (32).

### Brain metastasis

Brain metastasis, accounting for approximately 10%–30% of all cancer, occurs frequently in breast, kidney and lung cancers and melanoma and is usually observed at advanced stages. The blood-brain barrier (BBB) functions to stop CTCs from blood circulating to the brain. Cancer cells have to disrupt and pass through the BBB to invade the brain. Several studies have identified EVs as key factors that disrupt BBB permeability and promote brain metastases. miR-181c in breast cancer-derived exosomes degrade its target gene PDPK1 in endothelial cells. Inhibition of PDPK1 decreases the phosphorylation of cofilin and activates cofilin-induced modulation of actin dynamics, resulting in the destruction of the BBB (50).

Similarly, transference of miR-105 from breast cancer-derived exosomes to brain microvascular endothelial cells (BMECs) increases vascular permeability by reducing the tight junction protein ZO-1 (51). EVs derived from metastatic breast cancer also promote CTCs to cross the BBB system by transferring lncRNA GS1-600G8.5 to BMECs (52). A study by Morad et al. showed that transcytosis and the endothelial recycling endocytic pathway are involved in EV-mediated penetration of the BBB (53). Several studies have deciphered the reciprocal interactions between CTCs and the brain microenvironment. Gener et al. revealed that melanoma-secreted EVs created a hospitable PMN for tumorigenesis and metastasis by activating proinflammatory signaling in astrocytes (45). Tumor-derived EVs are taken up by astrocytes at the BBB in a Cdc42-dependent clathrin-independent carrier/GPI-AP-enriched compartment (CLIC/GEEC) endocytic pathway manner. The uptake of EVs containing miR-301a released from breast cancer by astrocytes alters the expression of ECM-remodeling proteins by downregulating TIMP-2 (54). Breast cancer derived-EVs carrying miR-1290 and miR-1246 activate astrocytes, which promote breast cancer stemness in the brain microenvironment. Moreover, EVs-miR-1290 promotes the progression of breast cancer brain metastasis through the FOXA2-CNTF signaling axis (55). Knockout of X-inactive-specific transcript preferentially enhances brain metastasis in breast cancer by releasing EVs containing miR-503. Mechanistically, exosomal miRNA-503 triggers M1–M2 polarization of microglia through STAT3 and NF-κB pathways. This M1–M2 conversion increases programmed cell death ligand 1 (PD-L1) expression in microglia, which contributes to the immunosuppressive microenvironment (56). These studies indicate that EVs secreted from metastatic brain tumors have immunomodulatory potential and can transfer astrocytes and microglia into tumor-supporting phenotypes. EVs-miR-122 from breast cancer cells increase glucose availability to cancer cells by suppressing glucose uptake by recipient astrocytes via downregulating glycolytic enzyme M2-pyruvate kinase (PKM2) and glucose transporter 1 (GLUT1) (57), indicating that EV-mediated reprogramming of glucose metabolism is another critical way to facilitate CTC colonization in the brain.

### Bone metastasis

Bone, second to lung and liver, is the third most frequent site of tumor metastasis. The most frequent primary tumors metastasizing to the bone include prostate, breast, bladder, renal, lung cancer and multiple myeloma. The imbalance between bone-resorbing osteoclasts and bone-forming osteoblasts activity occurs in bone metastasis, and osteoclastogenesis is induced. Bone marrow contains multiple cell types, including osteoblasts, myeloid cells, osteoclasts, adipocytes, fibroblasts and endothelial cells. Recent evidence has shown that communication between CTCs and bone cells mediated by EVs disturbs homeostatic bone and the formation of osteolytic and/or osteoblastic lesions (26). Zhang and colleagues found that multiple myeloma-derived EVs expressing miR-103a-3p inhibited osteogenesis in bone marrow-derived mesenchymal stem cells (BMSCs) and increased IL-6 secretion in multiple myeloma cells, resulting in impaired osteogenesis and exacerbated bone metastasis (61). LncRNA RUNX2-AS1 in myeloma cells packed into exosomes can transfer to mesenchymal stem cells (MSCs) and inhibit osteogenic differentiation in an EV-mediated lncRUNX2-AS1/Runx2-dependent manner (62). Under hypoxic conditions, amphiregulin (AREG) enriched in exosomes from multiple myeloma activates EGFR in pre-osteoclast. The uptake of exosomes by MSCs blocks osteoblast differentiation by activating the EGFR pathway and releasing pro-osteoclastogenic cytokine IL-8 (63). In non-small cell lung cancer (NSCLC), exosomal AREG increases the expression of RANKL, which subsequently increases the expression of osteoclastogenesis markers in pre-osteoclast by activating the EGFR pathway, promoting osteolytic bone metastasis (64). miR-21 in NSCLC-derived exosomes facilitates osteoclastogenesis via targeting programmed cell death 4 (PDCD4) (65). Breast cancer-secreted exosomal hsa-miR-940 promotes the osteogenic differentiation of MSCs by targeting ARHGAP1 and FAM134A (66). Henrich et al. reported the EV-mediated intercellular cross-talk between prostate cancer cells and bone marrow myeloid cells (BMMCs). Internalization of EVs by BMMCs activates NF-κB signaling, promotes osteoclast differentiation and decreases myeloid thrombospondin-1 expression, preparing for CTCs to implant and proliferate in bone (67). Phospholipase D (PLD) isoforms PLD1/2 promote tumor metastasis by catalyzing the hydrolysis of phosphatidylcholine to produce phosphatidic acid (PA). PLD1/2 present in EVs from prostate cancer cells activates osteoblast differentiation through enhancing extracellular signal-regulated kinase (ERK) 1/2 phosphorylation, tissue-nonspecific alkaline phosphatase activity and expression of osteogenic differentiation markers such as Alpl, Osx, Opn, Ocn and Dmp1, thus establishing a tumor-favoring microenvironment before bone metastasis (68). These findings validate the important role of EVs in bone microenvironment remodeling and PMN formation.

## The mechanism of EV-mediated PMN establishment

**Figure 2:**
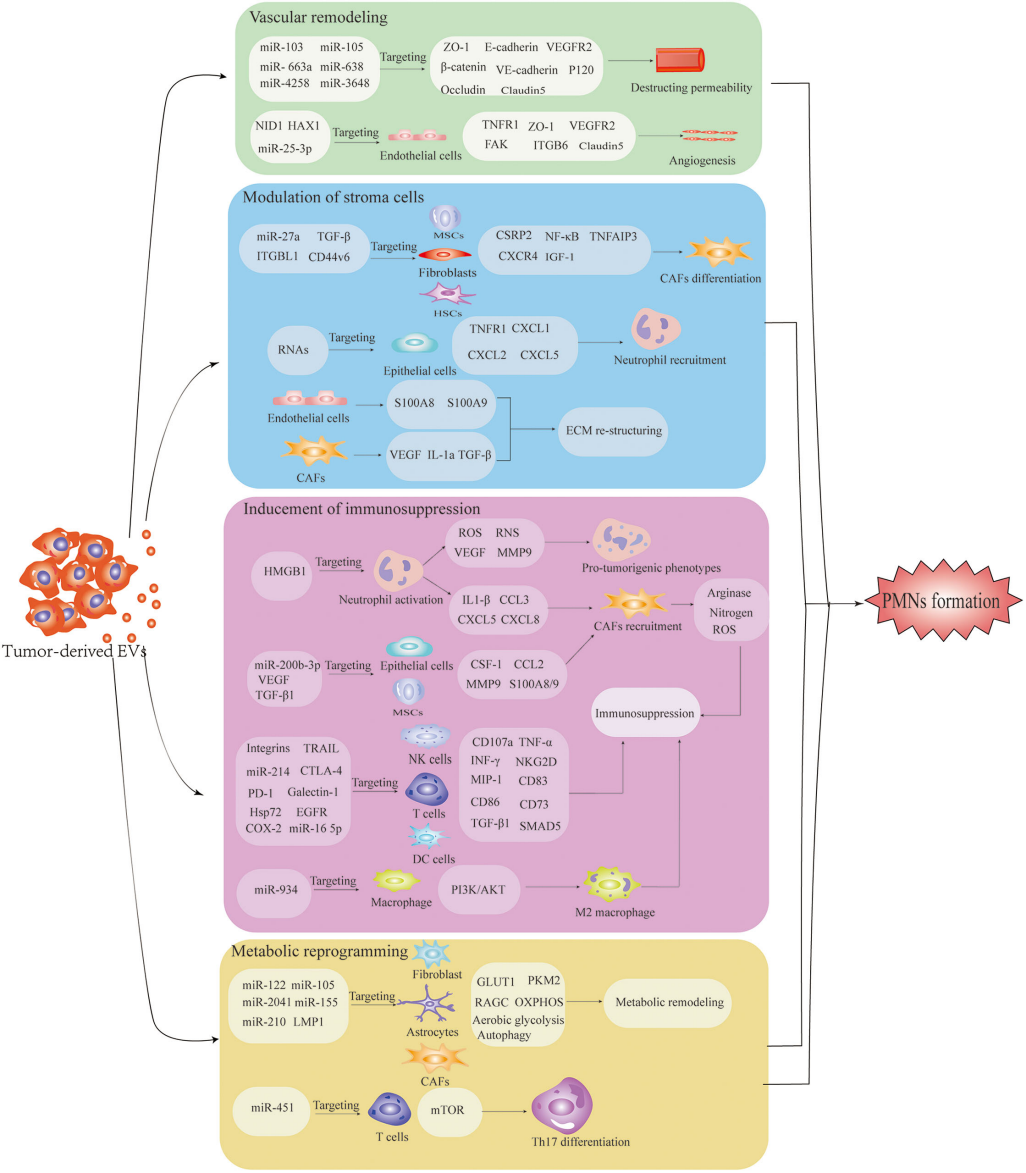
The underlying mechanism of pre-metastatic niche formation is induced by extracellular vesicles (EVs). EV-mediated interactions among tumor-recruited cells and local stromal cells, including endothelial cells, fibroblasts, astrocytes, epithelial cells, MSCs and immune cells, confer the characteristics of PMNs with organotropism, angiogenesis/vascular permeability, stroma reprogramming, immunosuppression and metabolic remodeling. Well-prepared PMNs favor the colonization and outgrowth of tumor cells, facilitating the establishment of PMNs.

Successful dissemination of tumors to secondary organs involves a series of processes, including local invasion, immune tolerance and evasion, permeation in systematic circulation and survival at metastatic sites (69). Growing evidence confirms the crucial role of EVs in cancer progression along the metastatic cascade (**Table 2**). The successful colonization of CTCs in various organs requires EV-mediated interactions between the tumor and microenvironment. The underlying mechanisms include 1) enhancement of angiogenesis and vascular remodeling, 2) stromal remodeling, 3) inhibitory effect of EVs on immune cells and 4) modification of the metabolic environment (**Figure 2**). In the following sections, we discuss the mechanisms of cancer-derived EVs relating to orchestrating PMN formation at secondary sites.

**Table 2:** The mechanism of EVs-mediated PMNs establishment.

Downstream effects	Donor cells	Cargo	Recipient cells	Specific mechanism	Refs.
Vascular remodeling	Breast cancer	miR-105	Endothelial cells	Inhibiting the tight junction protein ZO-1 to disrupt the vascular permeability	(63)
	HCC	miR-103	Endothelial cells	Destroying intercellular adhesion by destabilizing the E-cadherin/-catenin	(39)
	HCC	miR-638, miR- 663a, miR-3648, and miR-4258	Endothelial cells	Decreasing the expression of ZO-1 and VE-cadherin in endothelial cells to increase vascular permeability	(64)
	HCC	Nidogen 1	Endothelial cells	Enhanced angiogenesis and pulmonary endothelial permeability by increasing TNFR1	(65)
	CRC	miR-25-3p	Endothelial cells	Decreasing the expression of VEGFR2, ZO-1, occludin and Claudin5 in endothelial cells	(66)
	Nasopharyngeal carcinoma	HAX1	Endothelial cells	Activating the FAK in endothelial cells by increasing ITGB6 expression	(67)
Modulation of stroma cells	Gastric cancer	miR-27a	Fibroblasts	Inducing differentiation of fibroblasts into CAFs	(70)
	Gastric cancer	Bone morphogenetic proteins	Pericytes	Transforming pericytes into CAFs by activating PI3K/AKT and MEK/ERK	(51)
	Gastric cancer	TGF-	MSCs	Differentiating MSCs into CAFs by TGF/Smad	(71)
	CRC	Integrin beta-like 1	Fibroblasts	Activating fibroblasts via ITGBL1-TNFAIP3-NF-κB	(72)
	Lung cancer	Methylmalonic acid	Fibroblasts	Activating fibroblasts through the JAK/STAT3 and TGFβ activation	(73)
	Melanoma cells	snRNAs	Lung epithelial cells	Recruiting neutrophil via CXCL1, CXCL2, CXCL5, and CXCL12	(34)
	Pancreatic cancer	CD44v6/C1QBP	HSCs	Activating HSCs via phosphorylation of IGF-1	(74)
	Uveal melanoma	None provided	HSCs; Endothelial cells	Transforming HSCs and endothelial cells into a pro-fibrotic and pro-angiogenic phenotype	(75)
	Prostate cancer	miR-1227	CAFs	Enhancing migration of CAFs	(76)
Re-structuring ECM	Prostate cancer	Specific protein loaded in EVs	Specific organs	Increasing the activity of MMPs, fibronectin, collagen IV and number of CD11b^+^ cells	(77)
	Lung cancer	mutp53	Fibroblast	Promoting deposition of ECM via PODXL and Rab35	(78)
	Lung cancer	miR-29a-3p	Fibroblast	Inhibiting collagen I secretion to inhibit PMNs formation	(79)
	CRC	ANGPTL1	Kupffer cells	Decreasing the MMP9 expression via down-regulation of the JAK2-STAT3	(80)
Immune suppression	Gastric cancer	HMGB1	Neutrophil	Activating neutrophil through TLR4/NF-kB	(81)
	CRC	Exosomal-RNA	Neutrophil	Sustaining survival of neutrophils by inducing interleukin-1β expression	(82)
	Breast cancer	miR-200b-3p	AEC II	Enhancing expression of CSF-1, CCL2, S100A8/9 and MMP9 via AKT/NF-κB/CCL2	(83)
	CRC	miR-934	macrophage	Inducing M2 TAMs by activating PI3K/AKT and downregulating PTEN expression	(84)
	PDAC	Integrins	NK cells	Attenuating the NK cell cytotoxicity by down-regulation of NKG2D, CD107a, TNF-α, INF-γ, CD71 and CD98	(85)
	Head and neck cancers	COX-2, TGFβ-LAP, PD-1, CTLA-4 and TRAIL	NK cells; T cells	Inducing apoptosis of CD8^+^ T cells, inhibiting activity of NK cells and upregulating Treg functions.	(86)
	AML	CD33, CD34, CD117 and TGFβ1	T cells	Inducing apoptosis of CD8^+^ T cells and expanding Treg cells	(87)
	Head and neck cancers	Galectin-1	T cells	Transforming CD8^+^ T cells into a suppressed phenotype	(88)
	Melanoma	S100A8 and S100A9	Dendritic cell	Preventing DCs maturation by reducing expression of Flt3L, IL15, MIP-1α and MIP-1β	(89)
	Melanoma	PD-L1	T-cells	Effectively mading cytotoxic T-cells stagnated	(90, 91)
	Neck squamous carcinoma	PD-L1	T-cells	Inhibiting T-cell activity	(92)
	Prostate cancer and melanoma	PD-L1	T-cells	Decreased T cell activity	(93)
	Lung cancer	EGFR	Dendritic cells	Repressing antitumor immunity through inhibition of dendritic cells	(94)
	Lung cancer	EGFR	Macrophage	Reducing innate immunity by targeting the kinase MEKK2	(95)
	Thymoma, mammary carcinoma, and colon carcinoma	Hsp72	MDSCs	Suppressing activity and triggering the expansion of the MDSCs through activation of Stat3 and Erk	(96)
	Breast, hepatocellular, lung and pancreatic cancer	miR-214	Treg	Mediating Treg cell expansion by downregulating PTEN expression	(97)
	Breast cancer	miR-16 5p	Vδ1 T cells	Upregulating CD73 expression in Vδ1 T cells via activating TGF-β1/SMAD5 pathway	(98)
Metabolic remodeling	Breast cancer	miR-122	Brain astrocytes and lung fibroblasts	Inhibiting recipient cell glucose consumption through decreasing the expression of GLUT1 and PKM2	(49)
	Breast cancer	miR-105	CAFs	Enhancing CAFs glucose and glutamine metabolism to provide nutrients for cancer cells	(99)
	Gastric cancer	miR-451	T cells	Enhancing Th17 differentiation through activating mTOR activity	(100)
	Breast cancer	miR-105 and miR-204	Fibroblasts	Inhibiting amino acid -induced mTORC1 activity and protein synthesis in fibroblasts through targeting RAGC	(101)
	Nasopharyngeal carcinoma	LMP1	CAFs	Altering the metabolic status of CAFs to increase aerobic glycolysis and autophagy	(102)
	Melanoma	miR-155 and miR-210	Fibroblasts	Enhancing aerobic glycolysis and decrease OXPHOS	(103)

### Extracellular vesicles enhance angiogenesis and vascular remodeling in PMNs

A key characteristic of cancer biology is that cancer cells leave primary tissues and disseminate via circulating blood to the entire body. The vascular endothelial barrier is destroyed during early PMN formation. Induced angiogenesis, enhanced vascular permeability or disrupted vascular integrity within PMNs is an initial step for subsequent metastasis (70). Destruction of vascular integrity promotes the infiltration of cancer-derived EVs into PMNs. Wang et al. revealed that transfer of breast cancer-secreted miR-105 to endothelial cells mediated by EVs efficiently disrupted the vascular permeability by inhibiting the tight junction protein ZO-1, which facilitates breast cancer intravasation in primary tumor sites and extravasation at metastatic sites. Anti-miR-105 treatment suppresses the effect of exosomal miR-105 on niche adaptation (51). Similar effects are also found in HCC, CRC and breast cancer. In HCC, uptake of exosomal miR-103 by endothelial cells destroys intercellular adhesion by destabilizing the E-cadherin/β-catenin complex at the cell membrane by downregulating the expression of VE-cadherin, P120 and ZO-1 (60). Yokota et al. demonstrated that exosomal miRNAs (miR-638, miR- 663a, miR-3648 and miR-4258) released from HCC decreased the expression of ZO-1 and VE-cadherin in endothelial cells, thus increasing vascular permeability (71). Mao et al. found that nidogen 1 in HCC-derived EVs enhanced angiogenesis and pulmonary endothelial permeability to facilitate colonization of CTCs in the lung by activating fibroblasts to produce tumor necrosis factor receptor 1 (72). In CRC, the transfer of miR-25-3p from CRC cells to endothelial cells via exosomes promotes vascular permeability and angiogenesis at foreign sites. Mechanistically, exosomal miR-25-3p decreases the expression of VEGFR2, ZO-1, occludin and claudin5 in endothelial cells by targeting Krüppel-like factor 2 and Krüppel-like factor 4 (73). EVs rich in HAX1 promote metastasis and angiogenesis in nasopharyngeal carcinoma and are associated with blood vessel formation of endothelial cells. Concerning the mechanism, HAX1 activates the FAK pathway in endothelial cells by increasing the expression level of ITGB6 (104). These studies have confirmed that cancer cell-derived EVs are significantly involved in vascular permeability, promoting tumor cell invasion at primary sites and extravasating at distal sites.

### Extracellular vesicles are involved in stromal reprogramming in PMNs

#### Modulation of stroma cells

Metastatic tumor cells detached from a primary tumor establish a tumor-supportive microenvironment for CTC colonization by manipulating the microenvironment at secondary sites. Stromal cell remodeling driven by tumor-derived EVs, which develop a hypoxia and inflammation environment, is an important characteristic of PMNs (74). A critical mechanism underlying the formation of PMNs is the bilateral interaction between host stromal cells in distinct organs and the primary tumor. Stromal cells mainly include fibroblasts, glial cells, inflammatory cells, stellate cells, immune cells and pericytes. Tumor EVs are delivered to stromal cells and induce their differentiation into potent pro-tumorigenic phenotypes such as CAFs and TAMs (75). Among all the stromal cells, CAFs have a crucial effect on tumor initiation, progression and metastasis. Highly expressed miR-27a in exosomes isolated from gastric cancer induce differentiation of fibroblasts into CAFs and promote the metastasis of cancer cells via CSRP2 downregulation (76). Moreover, pericytes, known as progenitor cells that can differentiate into other cell types, can be transformed into CAFs by gastric cancer-derived exosomes by activating the PI3K/AKT and MEK/ERK pathways (105). MSCs, characterized by regenerative ability and multipotent capacity, can also be triggered to differentiate into CAFs by exosome-mediated TGF-β transference and TGF-β/Smad pathway activation in gastric cancer (106). Ji et al. demonstrated that CRC released integrin beta-like 1 (ITGBL1)-rich EVs to activate resident fibroblasts in lung and liver tissue through the ITGBL1-TNFAIP3-NF-κB signaling pathway. Activated fibroblasts enhance the secretion of proinflammatory cytokines such as IL-6 and IL-8 to induce the formation of PMNs (107). Simultaneously, JPJDR, a traditional Chinese medicine compound, inhibits fibroblast activation and reduces CRC liver metastasis by blocking the ITGBL1-rich EV-mediated TNFAIP3-NF-κB pathway (38). CAFs are the dominant cells in the tumor stroma that promote metastasis in primary tumors through secreting growth factors, chemokines, MMPs and ECM. Recent evidence indicates that CAFs in primary tumors also contribute to malignant initiation in distant organs through complex crosstalk with other cell types. Kong et al. found that CAF-derived EVs in salivary adenoid cystic carcinoma were involved in PMN formation in the lung by activating lung fibroblasts via the integrin α2β1/TGF-β signaling pathway (49). Li et al. reported that fibroblasts, activated by methylmalonic acid via reactive oxygen species (ROS) activating NF-κB and TGF-β pathway, secrete IL-6-loaded EVs that drive metastatic reprogramming through JAK/STAT3 and TGF-β signaling activation (108). Epithelial cells in the lung play an essential role in maintaining pulmonary homeostasis in response to pathogen- and injury-associated signals. Lung epithelial cells are also involved in local tumor metastasis by recognizing aberrant proinflammatory stimuli. Liu et al. showed that lung epithelial cells initiated neutrophil recruitment and lung metastatic niche formation by sensing tumor exosomal RNAs via TLR3. Activation of lung epithelial TLR3 by RNAs in tumor-derived exosomes via NF-kB and MAPK pathways increased the production of chemokines such as CXCL1, CXCL2, CXCL5, and CXCL12, leading to the recruitment and accumulation of neutrophils for PMN formation (58).

HSCs, a major source of αSMA-positive myofibroblasts and CAFs, are the main constituents in TME associated with liver metastasis. CRC cells activate and mediate the differentiation of HSCs to CAFs through the CXCR4/TGF-β1 axis (109). Activated HSCs enhance the expression of CCL20/CCR6 in CRC to form a CCL20/CCR6/ERK1/2/Elk-1/miR-181a-5p positive feedback loop between HSCs and CRC cells, leading to the reprogramming of TME and PMN formation (35). Pancreatic cancer-derived exosomes delivering the CD44v6/C1QBP complex promote live metastasis by activating HSCs and enhancing liver fibrosis via phosphorylation of insulin-like growth factor 1 signaling (110). Piquet et al. showed that EVs released by uveal melanoma (UM) modified HSCs in the liver. The uptake of UM-derived exosomes transformed HSCs to a myofibroblastic and contractile phenotype, inducing a pro-metastatic microenvironment (77). Caveolin-1-positive oncosomes from prostate cancer cells transfer miR-1227 to CAFs, transforming CAFs to form PMNs at secondary sites (78). Reducing stromal cell reactions by inhibiting COX-2 hinders the activation of HSCs and the accumulation of fibrillar collagen, providing novel targets for reducing the risk of metastasis (79).

#### Restructuring the ECM

The ECM embeds multiple cell types, including immune cells, endothelial cells and CAFs, in TME. Fibroblasts are the major cell type that secret high levels of collagen. Degradation of the ECM is a critical step in tumor invasion and metastasis. Many studies have shown that ECM remodeling is crucial for creating a microenvironment that enables colonization by CTCs (80, 111). ECM remodeling, including enhanced MMP activity and collagen, fibronectin and cytokines accumulation, is a primary process during PMN formation. Proinflammatory factors S100A8 and S100A9 produced by endothelial cells, expression of VEGF, IL-1a and TGF-β secreted by stromal cells and expression of HIF, VEGF and G-CSF induced by hypoxia promote the establishment of PMNs (112). The accumulation of fibronectin, MMPs, lysyl oxidase and other cytokines facilitates CTC adhesion and organ colonization (81, 82). Deep et al. showed that exosomes released by prostate cancer cells increased the activity of MMPs, fibronectin, collagen IV and several CD11b^+^ cells at distant organ sites (113). Fibronectin and collagen deposition enhance the recruitment of bone marrow-derived macrophages and neutrophils (21). In addition, exosomes from mut p53-expressing lung cancer cells activate fibroblasts to promote deposition of the ECM through PODXL- and Rab35-dependent mechanisms, generating a pro-invasive microenvironment (114). Therefore, downregulating the production of collagens may contribute to inhibiting lung metastasis. Yan et al. designed a lung-targeting liposomal nanovesicle carrying miR-29a-3p that significantly inhibited collagen I secretion by fibroblasts in lung tissue, thus relieving the formation of a pro-metastatic environment for CTCs (115). Overexpression of exosomal angiopoietin-like protein 1 in CRC attenuates liver metastasis by decreasing MMP9 expression via downregulation of the JAK2-STAT3 signaling pathway (83).

### Extracellular vesicles induce immunosuppression in the pre-metastatic niche

Immunosuppression is an important characteristic of PMNs. Building an immunosuppressive environment to protect circulating cancer cells from host immune attack is a key step for distant colonization and metastasis. Recent studies have shown that the microenvironment in a secondary organ is remodeled into an immunosuppressive state devoid of cancer cells (116). Immunosuppression reprogramming mediated by EVs in PMNs involves complex processes, including inhibition of immune cell response, inducing immune escape and regulating the network of immune molecules (117). EVs can also regulate the immune cell response, and consequently, developing such interactions has been proposed for inhibiting cancer progression. Stimulator of interferon genes (STING) agonists can activate anti-tumor immune responses, and engineering small-molecule STING agonists into EVs effectively stimulates antigen-presenting cells and enhances anti-tumor immunity (118). The first exosome phase I trial of dendritic cell (DC)-derived EV cancer vaccines (Dex) showed the safety of exosome administration, and an objective response in melanoma patients was observed (84). A phase II clinical trial of Dex confirmed the effectiveness of Dex to enhance natural killer (NK) and T cell antitumor responses in NSCLC (119). A variety of cellular components, such as MDSCs, Treg cells, neutrophils, macrophages, T cells and NK cells, are regulated by EVs to facilitate the process.

Tumor-associated EVs are involved in reprograming immune cells into immunosuppressive and pro-tumorigenic phenotypes. As the most abundant innate immune cell, neutrophils are a driving force for establishing inflammatory and immunosuppressive microenvironments. Zhang et al. confirmed that gastric cancer-derived exosomes delivered high mobility group box-1 (HMGB1) to neutrophils, which activates neutrophils through the interacting TLR4/NF-kB pathway. Blocking the HMGB1/TLR4/NF-κB interaction partially reversed exosome-induced neutrophil activation (120). Neutrophils can be mobilized to the lung by RNA-enriched tumor exosomes that activate alveolar epithelial TLR3 (58). Hwang et al. demonstrated that tumor stem-like cell-derived exosomal RNAs promote a pro-tumoral phenotype and sustain the survival of bone marrow-derived neutrophils by inducing interleukin-1β expression (121). Activated neutrophils induce genetic instability and carcinogenesis by producing reactive nitrogen species and ROS. VEGF and MMP9 released from neutrophils facilitate angiogenesis (85, 86). MDSCs are also recruited by chemokines, such as IL1-β, CXCL8, CXCL5, CCL2/17 and CCL3 secreted by neutrophils (87). MDSCs, a heterogeneous population of immature myeloid cells, play an indispensable role in establishing PMNs. Gu et al. showed that exosomal miR-200b-3p secreted from breast cancer “educated” alveolar epithelial type II cells (AEC II) to express colony-stimulating factor 1, CCL2, S100A8/9 and MMP9 through AKT/NF-κB/CCL2 cascades by binding to PTEN, thereby creating a suitable microenvironment for MDSC recruitment (88). MDSCs, with the high expression of arginase 1, nitrogen and reactive oxygen, suppress immune cells, such as T cells, B cells and NK cells (89, 122, 123). Moreover, macrophages, which can be converted into tumor-associated macrophages, are also crucial to immunosuppressive reprogramming in PMNs. Zhao et al. illustrated that CRC-derived exosomal miR-934 induced M2 macrophage polarization by activating the PI3K/AKT signaling pathway and downregulating PTEN expression. Polarized M2 macrophages promote liver metastasis through a CXCL13/CXCR5/NF-κB/p65/miR-934 positive feedback loop (124). Tumor-derived EVs also convert MSCs into pro-angiogenic and tumor-associated myofibroblasts by carrying VEGF and TGF-β1 and blocking immune cell anti-tumor activity (90). EVs derived from cancer cells inhibit the function of immune cells. NK cells are important innate immune cells that trigger anti-cancer immune responses and control metastatic dissemination by secreting PRF1- and GZMB-containing granules, IFN-γ and exposure of death receptor ligands such as FASLG and TRAIL (91, 92). Studies showed that EVs suppressed NK cell function directly. Integrins enriched in pancreatic cancer-derived EVs attenuate NK cell cytotoxicity by downregulating expression of NKG2D, CD107a, TNF-α, INF-γ, CD71 and CD98, inducing the phosphorylation of Smad2/3 and impairing glucose uptake (93). Moreover, Ludwig et al. found that exosomes from head and neck cancers carrying immunosuppressive proteins, such as COX-2, TGFβ-LAP, PD-1, CTLA-4 and TRAIL, suppress the cytotoxic activity of NK cells significantly by decreasing NKG2D expression (94). Similar findings were also reported from a study performed by Whiteside and colleagues (95). In this study, they further demonstrated that tumor-derived exosomes induced apoptosis of activated CD8^+^ T-cells and promoted the expansion of Treg cells. Maybruck et al. confirmed that galectin-1 in head and neck cancer cell-derived exosomes transformed CD8^+^ T cells into a suppressed phenotype (96). As tumor antigen-presenting cells, DCs are crucial for inducing initial immune responses (97). Maus et al. showed that melanoma-derived EVs prevented DC maturation by reducing the expression of Flt3L, IL15, MIP-1α and MIP-1β and inhibiting the expression of CD83 and CD86 (98).

Moreover, surface molecules of EVs can suppress immunity. PD-L1 and its receptor PD-1 have an immunosuppressive effect on activated T-cells to inhibit antitumor immune responses (99, 125). Convincing evidence revealed that EVs secreted from cancer cells deliver PD-L1, providing a new mechanism of EV-mediated immunosuppression. Recent studies demonstrated that exosomes from metastatic melanoma cells with high levels of PD-L1 effectively made cytotoxic T-cells stagnate, which protects circulating cancer cells from immune system attack (100, 101). PD-L1-positive exosomes from neck squamous carcinoma are associated with inhibiting T-cell activity and disease progression (102). Poggio et al. further confirmed that tumor exosomal PD-L1 decreased T cell activity, and exosomal PD-L1 inhibition induced an anti-tumor immune response (103). Besides PD-L1, other factors such as Hsp72, EGFR and RNAs carried by EVs exert an inhibited effect on immune cells. Tumor-derived exosomes deliver EGFR effectively to DCs and macrophages to suppress innate immunity and promote tumor progression (126, 127). Chalmin et al. reported that exosome-associated Hsp72 regulated suppressive activity and triggered the expansion of MDSCs through activation of Stat3 and ERK1/2 in a TLR2/MyD88-dependent manner (128). Some studies confirm the involvement of RNA in immunosuppression. Tumor-secreted miR-214 delivered by MVs mediates Treg cell expansion by downregulating PTEN expression (129). The breast cancer-derived exosomal miR-16 5p upregulates CD73 expression in Vδ1 T cells to exhibit an immunosuppressive effect by activating the TGF-β1/SMAD5 pathway (130).

#### EVS mediates modification of the metabolic environment in PMNs

Increasing evidence shows that metabolic reprogramming mediated by EVs is involved in PMN formation. Metabolites, including lactate, pyruvate, amino acids and fatty acids, accelerate the migration of cancer cells (131). Moreover, circulating cancer cells adapt their metabolic pathway to survive in a new environment. However, there is limited information showing the impact of EVs on the stromal cell metabolism in PMNs. Fong et al. found that uptake of breast cancer-derived exosomal miR-122 by niche cells (brain astrocytes and lung fibroblasts) in PMNs inhibits recipient cell glucose consumption by decreasing the expression of GLUT1 (also known as SLC2A1) and PKM2, thereby increasing nutrient utilization for cancer cells to facilitate metastasis (57). Exosomal miR-105 from cancer cells reprogram CAF metabolism to support cancer growth by conditioning the shared metabolic environment in an MYC-dependent manner. Exosomal miR-105 enhances CAF glucose and glutamine metabolism to provide nutrients for adjacent cancer cells under sufficient nutrients. When metabolic byproducts accumulate, and nutrient levels are reduced, CAFs detoxify lactic acid and ammonium by transforming these compounds into energy-rich metabolites (132). Moreover, miR-451 can be redistributed from cancer cells to infiltrated T cells by exosomes under glucose deprivation, enhancing Th17 differentiation in gastric cancer through activating mTOR activity (133). Cancer-derived EVs remodel amino acid (AA) metabolism in fibroblasts. Exosomal miR-105 and miR-204 from breast cancer inhibit AA-induced mTORC1 activity and protein synthesis in fibroblasts by targeting RAGC and altering the spectrum of *de novo* protein synthesis (134). Similarly, EVs from nasopharyngeal carcinoma packaged membrane protein 1 alter the metabolic status of CAFs. The reprogrammed CAFs increase aerobic glycolysis and autophagy to produce lactate and β-hydroxybutyrate (β-HB), providing intermediate metabolites to cancer cells for the tricarboxylic acid (TCA) cycle and oxidative phosphorylation (OXPHOS) (135). Melanoma-derived exosomes carrying miR-155 and miR-210 enhance aerobic glycolysis and decrease OXPHOS in human adult dermal fibroblasts, leading to extracellular acidification. The modulated metabolism of CAFs regulated by melanoma-derived exosomes promotes the establishment of PMNs (136). These studies demonstrate that targeting tumor-derived EVs to block stromal cell metabolic reprogramming may be an effective strategy to prevent metastasis.

## EV-mediated PMNs provide new ideas for targeted therapy

**Figure 3:**
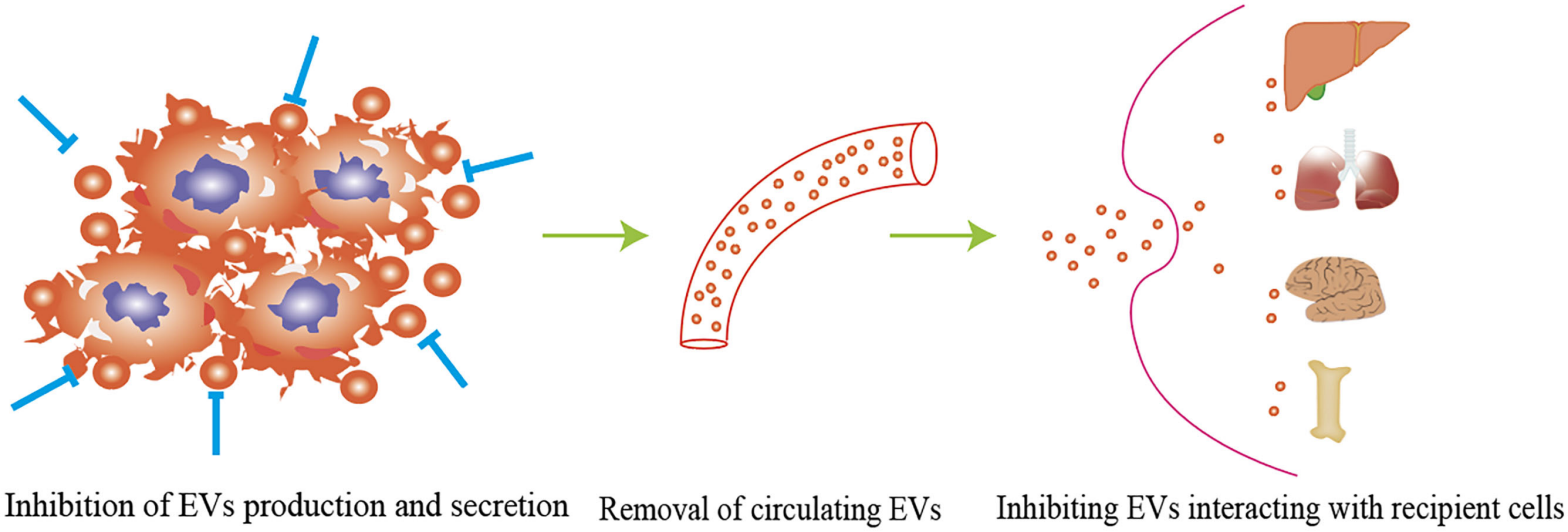
The strategy of targeting EVs to block EVs-mediated metastasis including inhibition of EVs production, removal of circulating EVs, and preventing EVs interacting with recipient cells.

The potential applications of EVs in cancer treatment can be categorized into the following classes: 1) early diagnostic biomarkers, 2) drug delivery, 3) chemoresistance targets, 4) cancer immunotherapy and 5) engineered EVs to prevent metastasis [12, 138]. As numerous studies have reported the role of diagnostic biomarkers, drug carriers and targeting therapy, this review mainly focuses on the potential application of EVs in inhibiting cancer metastasis. Various studies have evaluated the possibility of targeting EVs to block EV-mediated metastasis (**Table 3**). Studies have explored the potential approaches for inhibiting cancer metastasis through engineering EVs, and clinical trials have been conducted to evaluate the performance of EVs. Two phase I/IIa trials evaluating EVs incorporating STING agonists (exoSTING, NCT04592484) and IL-12 (exoIL-12, NCT05156229) were completed recently. The researchers evaluated the efficacy of exoSTING in treating advanced refractory solid tumors. Administering exoSTING was well tolerated, and tumor shrinkage was observed. ExoIL-12 gave favorable preliminary data in healthy subjects and early-stage cutaneous T-cell lymphoma patients (NCT05156229). In addition, EVs with KRASG12D targeting siRNA (exosomes) have shown therapeutic effects in some preclinical models. There is an ongoing phase I trial to evaluate the safety and efficacy of iExosomes in metastatic pancreatic cancer patients (NCT03608631). The antisense oligonucleotide of STAT6 enhances the reprogramming of tumor-associated macrophages to the M1 phenotype. The safety and efficacy of EVs with STAT6 antisense oligonucleotides (ASOs) (exoASO-STAT6) have been performed using patients with gastroenterological carcinoma (NCT05375604). As cancer therapeutic targets, EVs seem to be safe and well tolerated. Relevant clinical and preclinical validation studies will provide additional insight into the safety and efficacy of EV modalities. The strategies include inhibition of EV production, removal of circulating EVs and preventing the interaction of EVs with recipient cells (92) (**Figure 3**). Various approaches can be adopted to inhibit the release of EVs from cancer cells. As important modulators of EV biogenesis, Rab family proteins are suggested to be an ideal target to inhibit the release of EVs (137). Roma-Rodrigues and colleagues showed that the number of exosomes released from breast cancer cells decreased significantly by selective silencing Rab27a (138). Rab27a inhibition in mammary carcinoma cells leads to delayed primary tumor growth and lung dissemination by inhibiting EV secretion (139). Similarly, a study from Peinado showed that a Rab27a knockdown significantly reduced exosome secretion in melanoma, hindering tumor invasion and metastasis, suggesting that Rab27a is a potential target for preventing cancer metastasis (140). Ghoroghi et al. showed that GTPases of the Ral family facilitate the biogenesis of pro-metastatic EVs in breast cancer via phospholipase D1. RalA and RalB, members of the Ral family, reduced the expression of the adhesion molecule MCAM/CD146 in EVs to inhibit their dissemination and PMN development in lung tissue (141). In addition, selective inhibition of neutral sphingomyelinase 2, another regulator of EV release, in breast cancer reduced lung metastasis significantly (46). Overexpression of tubulin tyrosine ligase like 4, which codes for a cytoskeleton-associated protein, accelerates the velocity of secretory vesicles and multivesicular bodies, thereby promoting brain metastasis of breast cancers (142). A pharmaceutical approach using drugs that inhibit EV production has been widely studied. Several drugs identified to reduce EV biogenesis are GW4869, Tipifarnib, Imipramine, Manumycin A, Calpeptin and the antibiotic SFX (143). However, targeting EV release in preventing cancer metastasis remains controversial. Some undesired side effects may occur when suppressing EV biogenesis because EVs play critical roles in normal physiology and homeostasis.

**Table 3:** Therapeutic implications via targeting EVs to block EVs-mediated metastasis.

Therapy	Cancer type	Main finding	Refs.
Inhibiting EVs release	Breast cancer	Selective silencing of Rab27a decreases exosomes’ release	(127)
	Mammary carcinoma	Rab27a blockade leads to delayed primary tumor growth and lung dissemination	(128)
	Melanoma	Rab27a knockdown significantly reduced exosome secretion, hindering tumor invasion and metastasis	(129)
	Breast cancer	GTPases of the Ral family inhibits EVs dissemination and PMNs development in lung	(130)
	Breast cancer	Selective blockade of nSMase2 significantly reduces lung metastasis	(40)
	Breast cancers	Overexpression of TTLL4 promotes brain metastasis of breast cancers	(131)
Elimination of circulating EVs	Breast cancers	Aethlon Hemopurifier^®^ selectively capture and remove tumor-derived EVs to inhibit metastasis	(133)
	Breast cancers	Anti-CD9 or anti-CD63 antibodies is adopted to deplete circulating cancer-derived EVs to reduce lung and lymph node metastases	(134)
	Gastric cancer	Lactadherin is used to eliminate circulating EVs to prevents EVs-mediated pulmonary metastasis	(135)
Blocking the crosstalk between EVs and target cells	Melanoma	Reserpine inhibits the process of EVs uptake and disrupts EVs-mediated melanoma lung metastases	(136)

Eliminating circulating tumor-derived EVs can also inhibit PMN formation. A novel device termed Aethlon Hemopurifier^®^ has been proposed to selectively capture and remove tumor-derived EVs from the plasma of patients (144). The device evolves from kidney dialysis systems and is integrated with exosome-binding lectins and antibodies located in the outer capillary space of plasma filtration membranes. As the blood of the patient passes through the device, the circulating EVs are selectively eliminated without drug toxicity or interaction risks. A big challenge with this device is that there is no specific way to screen normal cell-derived exosomes and maintain intact conditions. Alternatively, a novel strategy using human-specific anti-CD9 or anti-CD63 antibodies has been adopted to deplete circulating cancer-derived EVs. Nishida-Aoki et al. reported that the antibody treatment significantly reduced lung and lymph node metastases without affecting primary tumor growth in breast cancer (145). The study further found that antibody-tagged EVs were preferentially internalized and eliminated by macrophages, suggesting that removing circulating EVs is a promising therapeutic option for inhibiting cancer metastasis. EVs from cancer cells displaying glucose chains, lipids and surface proteins can potentially be targeted by antibodies, which stimulate the elimination of cancer-derived EVs and inhibit their function. Similarly, lactadherin, which has been used to enhance the clearance of circulating EVs, has been shown to prevent EV-mediated disruption of endothelial integrity and inhibit pulmonary metastasis of gastric cancer (146). However, some uncertainties remain despite the promising therapeutic effect. Currently, there is a lack of specific biomarkers distinguishing between cancer-derived EVs and non-cancer-derived EVs. This low specificity will produce unwanted side effects and damage normal tissue when antibodies are used.

The interaction between tumor-derived EVs and target cells regulated by receptor-mediated endocytosis provides a possible strategy to block EV-mediated communication. Inhibiting the internalization of EVs by recipient cells is also an important approach to limiting EV-induced PMN formation. Ortiz et al. showed melanoma-derived EVs facilitate EV uptake and promote PMN establishment by downregulating the type I interferon receptor and cholesterol 25-hydroxylase. Reserpine, an anti-hypertensive drug, inhibited EV uptake and disrupted EV-mediated melanoma lung metastases (147). Another study reported that the internalization of exosomes derived from Epstein-Barr virus-infected cells by the recipient cells was mediated in a caveola-dependent endocytosis pathway. The process was inhibited significantly by the knockdown of caveolin-1 (148). However, conflicting results are obtained from another study (149). Hazan-Halevy et al. indicated that exosome released from mantle cell lymphoma uptake by target cells was not suppressed by caveolin-1 and clathrin knockdown but was regulated in a cholesterol-dependent pathway (149), suggesting that the mechanism underlying EV internalization is complicated and may be cell-type dependent.

Collectively, it is an efficient strategy to inhibit cancer metastasis by inhibiting the release of EVs by cancer cells, accelerating the elimination of circulating EVs and suppressing EV uptake by target cells. However, further research is needed to address the following concerns before translation into clinical applications: (1) complex composition in EVs, (2) uncertain biological functions and (3) the complex interaction with recipient cells.

## Conclusions and perspectives

Significant progress has been made in exploring the essential role of EVs in PMN formation since PMNs received attention in 2005 (150). Various studies have confirmed that tumor-derived EVs promote PMN development by enhancing angiogenesis, reprogramming stromal cells, restructuring the ECM, inducing immunosuppression and modifying the metabolic environment (151). Based on the critical role of EVs in mediating the formation of PMNs, targeting PMN-promoting EVs for metastasis inhibition is a feasible future option. In addition, identifying essential factors that regulate exosome biogenesis, circulation and endocytosis is also an approach for eliminating EVs and inhibiting the formation of PMNs. However, cancer and stromal cells in TME all secret EVs into circulation. Therefore, classification of generation, immunogenicity, organophilicity and biological circulation at the EV level is required when considering the wide variety of sources and complex functionality of EVs.

## Author contributions

SL: Project administration, Validation, Writing – original draft, Writing – review & editing. YL: Conceptualization, Data curation, Supervision, Validation, Writing – original draft. YZ: Data curation, Investigation, Writing – review & editing. XT: Formal analysis, Methodology, Project administration, Writing – review & editing. YD: Investigation, Methodology, Supervision, Writing – review & editing. YW: Project administration, Supervision, Validation, Writing – review & editing.
